# Alcohol-induced severe acute pancreatitis followed by hemolytic uremic syndrome managed with continuous renal replacement therapy

**DOI:** 10.1186/1471-2369-15-1

**Published:** 2014-01-06

**Authors:** Peng Fu, Ai-hong Yuan, Chun-hua Wang, Xin Li, Hai-yang Wu

**Affiliations:** 1 Department of Nephrology, Tongji Hospital, Tongji University, Alley 578, Xiangyin Road, Wujiaochang Town, 200433 Shanghai, China

**Keywords:** Acute pancreatitis, Acute renal failure, Continuous renal replacement therapy, Hemolytic-uremic syndrome

## Abstract

**Background:**

Acute kidney injury in patients with acute pancreatitis carries a poor prognosis. Hemolytic uremic syndrome (HUS) is characterized by non-immune hemolytic anemia, thrombocytopenia, and renal failure caused by platelet thrombi in the microcirculation of the kidney, and though rare in adults it is associated with high mortality and a high rate of chronic renal failure.

**Case presentation:**

Herein, we report a case of alcohol-induced acute pancreatitis in a 38-year-old Chinese female complicated by HUS. Her renal function progressively deteriorated in 2 days, and daily continuous renal replacement therapy (CRRT) was thus performed for a total of 13 treatments. She also received intermittent transfusions of fresh frozen plasma. Her renal failure was successfully managed, with subsequent return of normal renal function. She was discharged 1 month after admission and follow-up at 3 months revealed normal urea and creatinine.

**Conclusion:**

CRRT was shown to be useful for the treatment of HUS following acute pancreatitis. Prior case reports and our case should remind clinicians that HUS is a possible complication of acute pancreatitis. This study highlights the importance of early diagnosis and prompt initiation of CRRT to prevent mortality and improve outcomes.

## Background

The incidence of acute kidney injury in patients with acute pancreatitis ranges from 14-42%, and carries a poor prognosis [1]. Hemolytic uremic syndrome (HUS) as a result of alcoholic pancreatitis is seldom encountered, with only about 20 reported cases [1]. Herein, we report a case of alcohol-induced acute pancreatitis, followed by HUS which was successfully managed with continuous renal replacement therapy (CRRT), highlighting the importance of early diagnosis and prompt initiation of CRRT.

## Case presentation

A previously healthy 38-year-old female, non-smoker and non-alcoholic, was hospitalized following acute alcohol intoxication. She had drunk more than 500 ml of alcohol in about 2 hours and subsequently lost consciousness. Prior to losing consciousness she complained of severe epigastric pain and frequent vomiting. On admission, serum creatinine, blood urea nitrogen (BUN), hemoglobin, white blood cell (WBC) count, electrolytes, and liver function tests were within normal ranges. Serum amylase was 1728 IU/L (normal, < 100 IU/L). Ultrasound revealed the body and tail of the pancreas to be edematous, the biliary tree was not dilated, and there was sludge in the gallbladder. Abdominal computed tomography (CT) revealed an edematous pancreas with necrotic tissue in the left parieto-colic area. The diagnosis was alcohol-induced acute pancreatitis.

During the next 2 days her renal function progressively deteriorated, and her urine output decreased to < 400 ml per 24 hours. On the third day of admission her urea and creatinine were 12.8 mmol/L (normal range: 4.5-6.7 mmol/L) and 420 μmol/L (normal range: 50–110 μmol/L), respectively. Her hemoglobin level had dropped from 14.9 g/dL to 8.8 g/dL, with a reticulocyte count of 75,000/mm^3^ (normal range: 25,000-120,000/mm^3^), her platelet count was 32 × 10^9^/L (normal range: 100-300 × 10^9^/L), and haptoglobin was 0.05 g/L (normal range: 0.2-2.4 g/L). Peripheral blood smear revealed schistocytes, and the Coombs’ test was negative. The level of fibrin degradation products was normal, and her prothrombin time international normalized ratio (PT-INR) was 1.5-2.5. Early in the disease course plasma LDH is elevated in patient (856U/L, compared to normal LDH level: 35-123U/L), and LDH levels gradually decline with CRRT treatment and mitigation of pancreatitis. Her clinical symptoms of acute kidney injury and her treatment reaction were not consistent with a diagnosis of DIC. Renal biopsy was not performed because the patient refused invasive examinations. A diagnosis of HUS was made, and she was transferred to the intensive care unit (ICU).

She received daily continuous renal replacement therapy (CRRT) by hemofiltration on the third through the fifth hospital days, and then every other day for a total of 13 treatments. CRRT was administered at a dosage of 40 ml/kg/h for a duration of 6–8 hours, and the volume of fluid removed varied from 500 to 2500 ml. She also received intermittent transfusions of fresh frozen plasma. Her urine volume increased beginning of the fifth hospital day, and her serum amylase level returned to the normal range by hospital day 7. Her renal function progressively improved, and she was transferred from the ICU to the general ward on day 20. She was discharged 1 month after admission in good condition, and follow-up at 3 months revealed normal urea and creatinine (5.2 mmol/L and 69 μmol/L, respectively), and her platelet count was 129 × 10^9^/L. A year and 3months later, she gave birth to a healthy boy.

## Conclusions

Hemolytic uremic syndrome is characterized by non-immune hemolytic anemia, thrombocytopenia, and renal failure caused by platelet thrombi in the microcirculation of the kidney and other organs [2]. The disease is not uncommon in children, where approximately 80% of the cases are associated with *E. coli* O157:H7 infection and 80-90% of cases resolve without squelae [3]. HUS is rare in adults, and the overall prognosis in adults is poor with a reported mortality of up to 90% if not treated, and a rate of chronic renal failure of 40-60% [2,3].

The etiology of HUS following pancreatitis is not clearly understood, as there are only about 20 cases reported in the literature [1]. Our case and previously reported cases suggest that the acute inflammatory response to pancreatitis may trigger the onset of HUS [1]. Silva [4] hypothesized that the release of inflammatory mediators such as tumor necrosis factor (TNF)-alpha and interleukin (IL)-1 during an acute episode of pancreatitis may induce widespread vascular endothelial injury. It has also been suggested that modification of circulating von Willebrand factor (VWF) by pancreatic proteases leads to platelet aggregation [4,5]. The release of ultralarge VWF multimers from endothelial cells during inflammation, and the inhibition of the VWF cleaving protease ADAMTS-13 by cytokines have also been suggested as a contributory mechanism of HUS [6].

The treatment of HUS following acute pancreatitis is primarily supportive. Fresh frozen plasma infusion may improve the renal outcome in adult HUS [2], and exchange plasmapheresis has been reported to be an effective treatment with a response rate of 79% [7]. However, in patients with relapsing or refractory HUS with or without ADAMTS-13 deficiency, plasma exchange is often unsuccessful [8]. Rituximab, a chimeric monoclonal antibody directed against CD20 antigen expressed by B lymphocytes, has shown promise as a treatment option [8,9]. At the time the patient was seen and treated plasmapheresis was not available (treatment was carried out before dawn and CRRT was performed in the emergency department), and because CRRT therapy was very effective performing plasmapheresis was not considered necessary.

In renal replacement therapy (RRT) performed via hemofiltration, solute movement is governed by convection and unlike in dialysis a dialysate is not used [10]. Positive hydrostatic pressure causes water and solutes to migrate across a filter membrane from the blood to the filtrate compartment, where it is drained. An isotonic replacement fluid is added to the blood to replace fluid volume and electrolytes. Treatments can be given intermittently, or continuously. CRRT has proven to be particularly useful in the treatment of renal failure as it can help in restoration of acid–base imbalances and electrolyte abnormalities, maintain hemodynamic stability, and allows the adjustment drug dosages to prevent drug accumulation and overdose [10]. In addition, it can remove metabolic waste products and clear inflammatory mediators such as IL-1, IL-6, IL-8. It is possible that removal of inflammatory mediators by the rapid initiation of CRRT contributed to the recovery of our patient, though we have no clear evidence to support this hypothesis.

In summary, we have presented a case of adult HUS secondary to acute pancreatitis successfully managed by prompt diagnosis and rapid initiation of CRRT via hemofiltration before severe kidney injury or renal failure had occurred. Prior case reports and our case should remind clinicians that HUS is a possible complication of acute pancreatitis. Early institution of CRRT may be an effective treatment to prevent mortality and improve outcomes.

## Consent

Written informed consent was obtained from the patient for publication of this Case report. A copy of the written consent is available for review by the Editor of this journal.

## Competing interests

The authors declare that they have no competing interests.

## Authors’ contributions

PF: guarantor of integrity of the entire study; study concepts; study design; definition of intellectual content; data analysis; statistical analysis; manuscript preparation; manuscript editing; manuscript review. AHY: literature research. CHW: clinical studies. XL: clinical studies. HYW: data acquisition. All authors read and approved the final manuscript.

## Pre-publication history

The pre-publication history for this paper can be accessed here:

http://www.biomedcentral.com/1471-2369/15/1/prepub
